# Juanbi Tang Attenuates Intervertebral Disc Degeneration by Suppressing p38MAPK/NF‐κB Signaling and Activating Atg5‐Dependent Autophagy to Promote Macrophage M2 Polarization: In Vivo and In Vitro Evidence

**DOI:** 10.1002/iid3.70498

**Published:** 2026-09-10

**Authors:** Ye‐Hui Wang, Wei Hou, Guo‐Sheng Tang, Xuan‐Geng Deng, Si‐Mao Song, Wei Cui, Yang Ye, Yang Yu

**Affiliations:** ^1^ Department of Spine and Neurosurgery Sichuan Province Orthopedic Hospital Chengdu China; ^2^ Hospital of Chengdu University of Traditional Chinese Medicine Chengdu China

**Keywords:** Atg5, Autophagy, Intervertebral disc degeneration, Juanbi Tang, Macrophage polarization, NF‐κB, Nucleus pulposus, p38MAPK, Polypharmacology, Traditional Chinese medicine

## Abstract

**Background:**

Intervertebral disc degeneration (IDD) is driven by macrophage‐mediated inflammation involving the p38MAPK/NF‐κB signaling pathway and impaired autophagic flux. Juanbi Tang (JBT), a classical eleven‐herb Chinese formula documented since 1732 CE for the treatment of Bi syndrome (a traditional diagnosis encompassing painful musculoskeletal obstruction conceptually corresponding to degenerative spinal conditions), demonstrates clinical anti‐inflammatory activity; however, its molecular mechanism in IDD remains uncharacterized.

**Methods:**

JBT chemical composition was characterized by HPLC fingerprinting with osthole as quantitative reference standard (inter‐batch RSD 2.3%) in accordance with the 2020 Chinese Pharmacopoeia, and quality control was extended beyond a single marker constituent by multi‐component fingerprint similarity analysis using the cosine angle and correlation coefficient methods. A male Sprague–Dawley rat annular puncture IDD model (five groups: Sham, IDD, IDD + JBT, IDD + 3‐methyladenine [3‐MA], and IDD + JBT + 3‐MA) was evaluated by MRI, CT, Safranin O–Fast Green, Masson trichrome and hematoxylin and eosin staining. LPS‐stimulated (1 μg/mL) primary rat nucleus pulposus (NP) cells and murine RAW264.7 macrophages — including Atg5‐overexpressing (Atg5‐OE) and knockdown (Atg5‐KD) variants — were used for in vitro mechanistic studies. Endpoints included apoptosis, mitochondrial membrane potential (ΔΨm), reactive oxygen species (ROS), macrophage M1/M2 polarization, ELISA cytokine quantification and Western blot pathway analysis.

**Results:**

In vivo, JBT significantly improved Pfirrmann grade, disc height index, Safranin O staining intensity, Masson‐stained collagen content and Thompson grade, with concomitant suppression of serum TNF‐α and restoration of IL‐10; all benefits were partially abrogated by 3‐MA co‐administration. JBT suppressed p‐p38MAPK and p‐NF‐κB p65 and restored the COL II/MMP‐13 balance in disc tissue. In NP cells, JBT reduced total apoptosis from 29.1% to 14.5%, restored ΔΨm, attenuated ROS accumulation, reversed the Bax/Bcl‐2 ratio, and activated autophagy (LC3‐II ↑ , p62 ↓ ). In RAW264.7 macrophages, JBT promoted M2 polarization (CD206^+^: 28.1% to 49.7%) and suppressed M1 polarization (CD197^+^: 52.4% to 18.9%) under LPS stimulation, with concurrent autophagy activation. Western blot analysis across normal, Atg5‐OE and Atg5‐KD macrophages demonstrated that Atg5 expression level bidirectionally modulates p62 clearance and consequent NF‐κB suppression, consistent with the autophagy–p62–NF‐κB regulatory module contributing to JBT's pharmacological activity.

**Conclusions:**

JBT attenuates IDD through coordinated suppression of macrophage‐driven inflammation and activation of autophagy, providing mechanistic support for its clinical application in musculoskeletal disease.

## Graphical abstract

Graphical Abstract. Juanbi Tang (JBT) suppresses the p38MAPK/NF‐κB signaling pathway in disc‐infiltrating macrophages, simultaneously activating Atg5‐dependent autophagy. Autophagic degradation of p62/SQSTM1 consolidates NF‐κB inhibition through an autophagy–p62–NF‐κB regulatory module, facilitating macrophage M1‐to‐M2 polarization. The anti‐inflammatory disc microenvironment thereby established attenuates nucleus pulposus cell apoptosis, restores mitochondrial homeostasis, and preserves disc structural integrity.

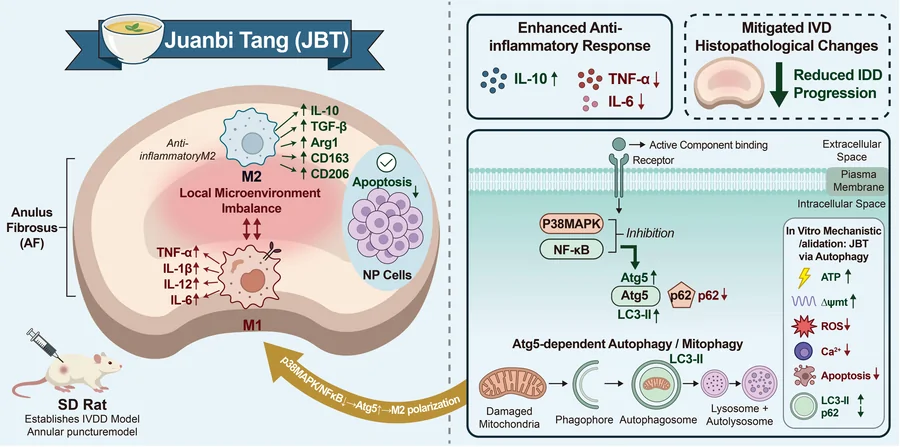

**Abbreviations**
3‐MA: 3‐methyladenine
COL II: type II collagen
CT: computed tomography
DHI: disc height index
ECM: extracellular matrix
ELISA: enzyme‐linked immunosorbent assay
HE: hematoxylin and eosin
HPLC: high‐performance liquid chromatography
IDD: intervertebral disc degeneration
JBT: Juanbi Tang
JC‐1: mitochondrial membrane potential dye
LPS: lipopolysaccharide
MMP‐13: matrix metalloproteinase‐13
MRI: magnetic resonance imaging
NF‐κB: nuclear factor kappa B
NP: nucleus pulposus
p38MAPK: p38 mitogen‐activated protein kinase
ROS: reactive oxygen species
RPA: relative peak area
RT‐qPCR: reverse‐transcription quantitative PCR
SO: Safranin O
TCM: traditional Chinese medicine
ΔΨm: mitochondrial membrane potential

## 1 Introduction

Intervertebral disc degeneration (IDD) is a major cause of chronic low back pain and disability worldwide [1, 2]. The degenerating disc is characterized by progressive nucleus pulposus (NP) cell apoptosis, extracellular matrix (ECM) degradation, annular disruption, and establishment of a self‐perpetuating pro‐inflammatory microenvironment. Current therapeutic strategies—analgesics, rehabilitation, and surgery—address symptomatic burden without modifying disease progression. This unmet need for disease‐modifying therapy has driven intensive investigation into the immunopathological mechanisms governing IDD.

Macrophage polarization has emerged as a central determinant of IDD trajectory. M1‐polarized macrophages, identified by CD197 surface expression, secrete TNF‐α, IL‐1β, and MMP‐13, driving NP cell apoptosis and ECM catabolism. M2‐polarized macrophages, identified by CD206 expression, produce IL‐10 and TGF‐β, promoting tissue homeostasis and NP cell survival [3, 4]. Pharmacological promotion of the M1‐to‐M2 transition represents a mechanistically rational therapeutic approach in IDD [5].

The p38MAPK/NF‐κB signaling pathway is a central convergence node for diverse inflammatory stimuli in the disc microenvironment. Mechanical stress, damage‐associated molecular patterns, and pro‐inflammatory cytokines activate p38MAPK, which phosphorylates NF‐κB p65 and drives transcription of MMP‐13, TNF‐α, and IL‐1β [6, 7]. Autophagy, mediated by Atg5‐dependent autophagosome formation, has recently been recognized as a critical regulator of macrophage polarization through selective degradation of p62/SQSTM1, a scaffold protein sustaining tonic NF‐κB activation. Autophagic p62 clearance thus forms a regulatory module that consolidates NF‐κB inhibition and lowers the M1 polarization threshold [8, 9].

Traditional Chinese medicine (TCM) offers a rich source of multi‐target agents suited to the complex immunopathology of IDD. Juanbi Tang (JBT) is a classical formula first documented in Yi Xue Xin Wu (“Medical Reflections”) by Cheng Guopeng of the Qing dynasty (1732 CE), prescribed for Bi syndrome—painful musculoskeletal obstruction attributed to wind, cold, and dampness pathogenic factors. In classical TCM nosology, lumbar Bi syndrome encompasses the clinical features of IDD: local inflammation, structural rigidity, impaired circulation, and chronic pain. JBT has been used in continuous clinical practice for degenerative spinal and joint conditions, with pharmacological studies documenting anti‐inflammatory, analgesic, and tissue‐protective activities [10].

JBT comprises eleven herbs organized by the jun‐chen‐zuo‐shi (sovereign‐minister‐assistant‐courier) compositional principle. Notopterygium incisum Ting ex H.T. Chang (Qianghuo, 10 g) and Angelica pubescens Maxim. f. biserrata Shan et Yuan (Duhuo, 10 g) serve as sovereign herbs with primary anti‐inflammatory and analgesic activity. Cinnamomum cassia Presl (Rougui, 10 g), Gentiana macrophylla Pall. (Qinjiao, 10 g), Ligusticum chuanxiong Hort. (Chuanxiong, 6 g), and Angelica sinensis (Oliv.) Diels (Danggui, 10 g) serve as minister herbs augmenting circulation and attenuating matrix catabolism. Piper kadsura (Choisy) Ohwi (Haifengteng, 10 g) and Morus alba L. (Sangzhi, 10 g) function as assistant herbs reinforcing anti‐inflammatory activity. Aucklandia lappa Decne. (Muxiang, 6 g) and Boswellia sacra Flueck. (Ruxiang, 6 g) serve as additional assistant herbs; boswellic acids from Boswellia sacra Flueck. are established NF‐κB inhibitors [11]. Glycyrrhiza uralensis Fisch. ex DC. (Gancao, 5 g) serves as the courier herb harmonizing formula actions. This compositional architecture achieves polypharmacological coverage of multiple inflammatory pathways simultaneously.

HPLC‐Q‐TOF analysis of JBT has identified 44 chemical constituents across all eleven herbs, including coumarins, phenylpropanoids, flavonoids, iridoids, and terpenoids [10]. Osthole, the mandated quality‐control marker of Angelica pubescens Maxim. f. biserrata Shan et Yuan per the 2020 Chinese Pharmacopoeia, has been independently reported to suppress p38MAPK and NF‐κB signaling in musculoskeletal tissues [12, 13]. The formula's full therapeutic effect reflects integrated polypharmacological action across all eleven constituent herbs.

In the present study, we systematically investigated the therapeutic efficacy and mechanistic basis of JBT in IDD using a rat annular puncture model, LPS‐stimulated NP cell cultures, and RAW264.7 macrophage systems with Atg5 genetic manipulation, to establish the mechanistic relationships linking p38MAPK/NF‐κB suppression, autophagy activation, and M2 macrophage polarization to disc structural preservation.

## 2 Materials and Methods

### 2.1 Preparation and Chemical Characterization of Juanbi Tang

JBT was prepared from eleven medicinal herbs authenticated per the 2020 Chinese Pharmacopoeia (voucher specimen information in Supplementary Table S2): Notopterygium incisum Ting ex H.T. Chang (10 g), Angelica pubescens Maxim. f. biserrata Shan et Yuan (10 g), Cinnamomum cassia Presl (10 g), Gentiana macrophylla Pall. (10 g), Ligusticum chuanxiong Hort. (6 g), Angelica sinensis (Oliv.) Diels (10 g), Piper kadsura (Choisy) Ohwi (10 g), Morus alba L. (10 g), Aucklandia lappa Decne. (6 g), Boswellia sacra Flueck. (6 g), and Glycyrrhiza uralensis Fisch. ex DC. (5 g). Herbs were soaked in 1,000 mL purified water for 60 min, decocted twice (rolling boil, 30 min per decoction), filtered through 200‐mesh cloth, and vacuum‐concentrated (≤ 50°C) to 77.5 mL (1.2 g/mL crude drug). Preparations were stored at 4°C.

**Figure 1:**
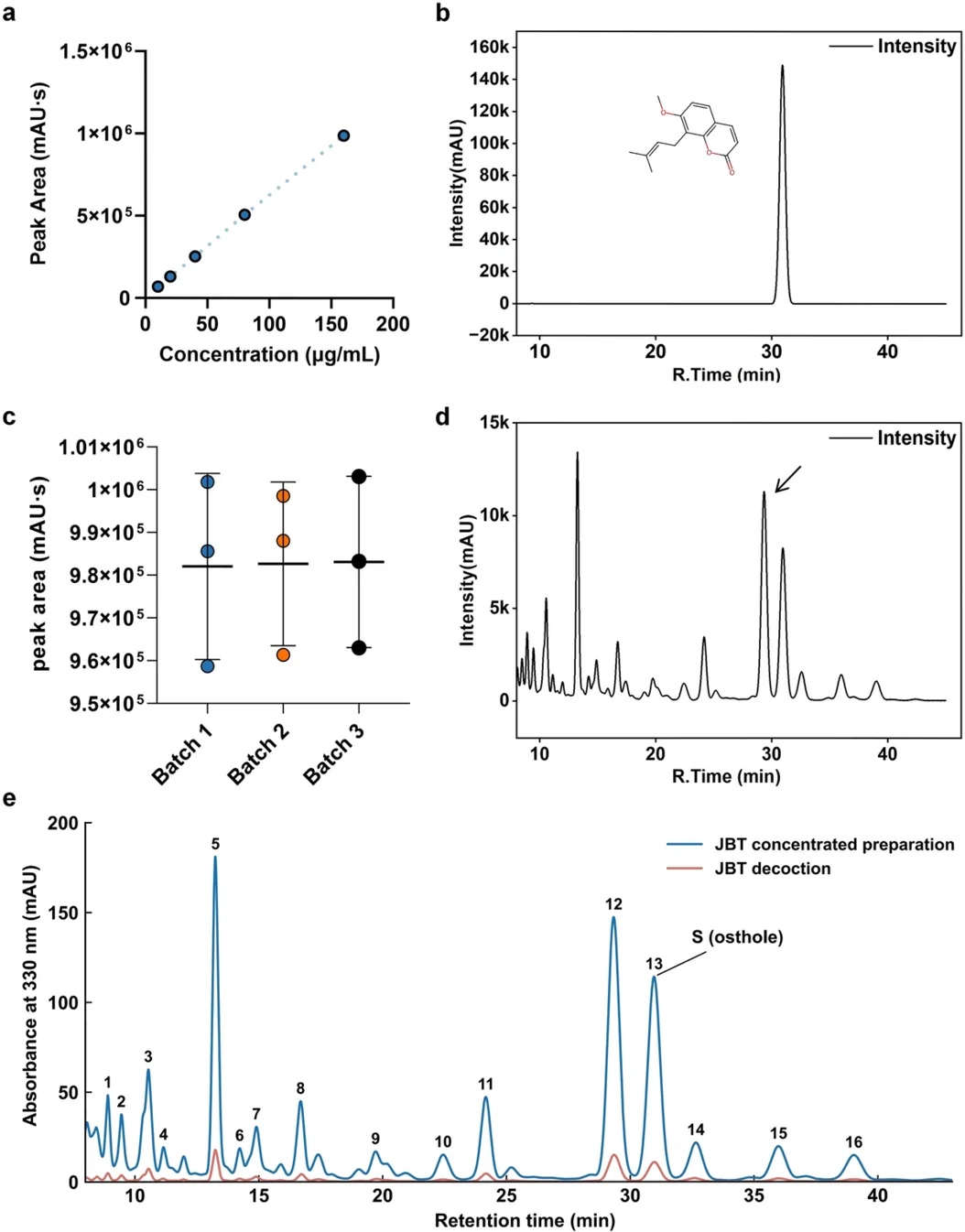
HPLC fingerprinting and batch quality control of Juanbi Tang (JBT). (a) Osthole standard curve (20–160 μg/mL; *R* ^2^ > 0.999), confirming quantitative linearity. (b) Osthole reference standard chromatogram (160 μg/mL; retention time 30.927 min; USP plate number 16,188). (c) Batch reproducibility of osthole peak area across three independent JBT preparations (n = 3 injections per batch); horizontal bars indicate mean ± SD; inter‐batch RSD = 2.3% (acceptance threshold < 5%). (d) Representative HPLC fingerprint of JBT concentrated preparation (1.2 g/mL crude drug equivalent); osthole identified at 30.962 min (arrow); multi‐peak profile is consistent with the 44‐constituent chemical composition previously reported by Wang et al. [10]. (e) Overlaid HPLC fingerprints of the JBT decoction and the vacuum‐concentrated preparation over the 8–43 min window. Sixteen common peaks were resolved and are numbered sequentially; the osthole peak (peak 13, 30.96 min) was designated as the reference (S) peak. Similarity between the two profiles was 0.9994 by the cosine angle method and 0.9989 by the correlation coefficient method; the RSD of relative peak area across all sixteen common peaks was below 3.3% on replicate analysis. The region before 8 min was excluded because the response of early‐eluting peaks approached the upper limit of detector linearity. Detection: 330 nm PDA; column: Agilent TC‐C18 (250 × 4.6 mm, 5 μm).

HPLC fingerprinting was performed using osthole as quantitative reference standard, consistent with its designation as the mandated quality‐control marker for Angelica pubescens Maxim. f. biserrata Shan et Yuan in the 2020 Chinese Pharmacopoeia. A standard curve was constructed across the concentration range 20–160 μg/mL to confirm linearity (Figure 1a). Chromatographic separation: Agilent TC‐C18 column (250 × 4.6 mm, 5 μm); acetonitrile (A)–water (B) gradient elution: 0–5 min 20% A; 5–25 min 20–65% A; 25–35 min 65–80% A; 35–40 min 80% A; 40–45 min 80–20% A; detection 330 nm (PDA); column temperature 35°C; flow rate 1.0 mL/min; injection 10 μL. Osthole content was calculated by the external standard method. Batch reproducibility was assessed across three independent preparations by calculating the relative standard deviation (RSD) of the osthole peak area.

To extend quality control beyond a single marker constituent, a multi‐component fingerprint similarity analysis was additionally performed. Chromatograms were exported as digitized traces and evaluated over the 8–43 min window; the region before 8 min was excluded because the response of early‐eluting peaks in the concentrated preparation approached the upper limit of detector linearity. Peaks were detected after Savitzky–Golay smoothing using a detection threshold of 0.5% of the maximum peak height, and peak areas were integrated against a linear baseline drawn between peak start and end points. Sixteen common peaks were assigned across preparations, with the osthole peak designated as the reference (S) peak, and the relative peak area (RPA) of each common peak was calculated against the S peak. Similarity of the resulting RPA vectors was computed by both the cosine angle method and the correlation coefficient method, in accordance with the principles of the Chinese Pharmacopoeia Commission chromatographic fingerprint similarity evaluation system.

The major pharmacologically active constituents of JBT as characterized by HPLC‐Q‐TOF [10] include: osthole (from Angelica pubescens Maxim. f. biserrata Shan et Yuan), notopterol (from Notopterygium incisum Ting ex H.T. Chang), ferulic acid and ligustilide (from Ligusticum chuanxiong Hort.), gentiopicroside (from Gentiana macrophylla Pall.), cinnamaldehyde (from Cinnamomum cassia Presl), glycyrrhizin and liquiritin (from Glycyrrhiza uralensis Fisch. ex DC.), and acetyl‐11‐keto‐β‐boswellic acid (AKBA; from Boswellia sacra Flueck.) [11, 14, 15].

### 2.2 Animal Model and Experimental Groups

All experiments were reviewed and approved by the Animal Ethics Committee of West China Hospital, Sichuan University (No. 20231116001; 16 November, 2023) and conducted in accordance with the internationally accepted principles for laboratory animal use and care, including the European Community guidelines (EEC Directive of 1986; 86/609/EEC) and the US guidelines (NIH publication #85‐23, revised 1985). Animal procedures were performed at the animal experimental platform of West China Hospital, Sichuan University. Animal experiments were designed and reported in accordance with the ARRIVE 2.0 guidelines. Male Sprague–Dawley rats (7–8 weeks old, 200–220 g) were purchased from Chengdu Dossy Experimental Animals Co. Ltd. (Chengdu, China). All animals were specific‐pathogen‐free, wild‐type, and had not undergone any previous experimental procedure. Rats were housed three per cage in standard polycarbonate cages with corncob bedding under controlled conditions (22 ± 2°C, relative humidity 55 ± 10%, 12 h light/dark cycle), with standard rodent chow and water available ad libitum; nesting material was provided as environmental enrichment. Animals were acclimatized for 1 week before the experiment. IDD was induced by full‐thickness annular puncture at Co6/7 and Co7/8 under sterile conditions [16]. All surgical procedures were performed under inhalational anesthesia with isoflurane, and a prophylactic antibiotic was administered postoperatively. Twenty‐one animals per group were enrolled in order to provide independent, non‐overlapping tissue samples for the separate endpoint analyses and to accommodate potential attrition. Animals (*n* = 105 total) were randomized into five groups (*n* = 21/group): Sham, IDD, IDD + JBT, IDD + 3‐MA, and IDD + JBT + 3‐MA, using a random number sequence generated in Microsoft Excel (RAND function). Cages were assigned to rack positions without regard to group allocation, and surgery, treatment administration, and outcome measurement were not performed in group order; no further measures were taken to control potential confounders. Within each group, animals were subsequently allocated to non‐overlapping endpoint subsets—imaging and histology, Western blot, and ELISA—and *n* = 5 animals per group were analyzed for each endpoint reported; the remaining animals were used for additional tissue sampling and were retained as replacements for attrition. Intervention began 24 h post‐surgery and continued 4 weeks. Sham and IDD groups received distilled water by gavage. The IDD + JBT group received JBT concentrate by oral gavage twice daily (total 7.5 mL/kg/day), corresponding to approximately 9.0 g crude drug/kg/day by body surface area conversion from the clinical human dose of the formula (93 g crude drug per day for a 60 kg adult). This dose was selected on the basis of published dose‐ranging data demonstrating dose‐dependent anti‐inflammatory effects of JBT in a collagen‐induced arthritis model [10], and is consistent with the effective therapeutic dose range validated in that study. Full dose–response characterization in the rat annular puncture IDD model is planned as a subsequent study. The IDD + 3‐MA group received 3‐methyladenine (Sigma‐Aldrich; 10 mg/kg, twice weekly i.p.). The IDD + JBT + 3‐MA group received both interventions. Animals were monitored daily for body weight, wound healing, and general activity. Criteria for early termination were body weight loss exceeding 20%, persistent wound infection, hindlimb paralysis, or inability to feed. No animal died, developed wound infection, or met the criteria for early termination during the 4‐week study period; no unexpected adverse events occurred, and no animals or data points were excluded from any analysis. At 4 weeks, animals were euthanized under isoflurane anesthesia.

### 2.3 Imaging and Histological Assessment

MRI T2‐weighted sagittal images were acquired at 4 weeks; NP signal intensity was normalized to adjacent paraspinal muscle (ImageJ). Pfirrmann grading (I–V) was assessed by two blinded observers [17]. CT sagittal reconstructions were used to calculate the disc height index (DHI). Histological sections (5 μm paraffin) were stained with Safranin O–Fast Green (proteoglycan content), Masson trichrome (collagen fiber organization), and HE (overall morphology). Proteoglycan content was semi‐quantified by mean optical density (MOD, Image‐Pro Plus 6.0). Masson‐positive area fraction was quantified by ImageJ. Modified Thompson histological grading was performed by two blinded observers [18]. Throughout, the investigator who performed the surgery and administered the interventions was aware of group allocation; all imaging and histological outcome assessments were performed by observers blinded to group allocation; data analysis was performed with group identity known.

### 2.4 Primary Nucleus Pulposus Cell Culture

Primary rat NP cells were isolated from caudal discs under sterile conditions and cultured in DMEM/F‐12 with 10% FBS (passage 3 used throughout). Three groups: Control, LPS (1 μg/mL, 24 h), and LPS + JBT (LPS 1 μg/mL + JBT 50 μg/mL, 24 h). LPS concentration (1 μg/mL) was selected based on published evidence of reliable NP cell inflammatory induction at this dose with preserved viability (> 80%, confirmed by preliminary CCK‐8 assay) [19].

### 2.5 Macrophage Culture and Atg5 Genetic Manipulation

Murine RAW264.7 macrophages were maintained in DMEM/10% FBS/1× P/S. Atg5 overexpression (Atg5‐OE) was achieved by Lipofectamine 2000‐mediated transfection of pcDNA3.1‐Atg5; empty vector served as control. Atg5 knockdown (Atg5‐KD) was achieved using siRNA003‐Atg5; scrambled siRNA served as control. Transfection efficiency was confirmed by RT‐qPCR at 48 h (thresholds: OE > 3‐fold upregulation vs. normal; KD < 30% residual expression). For polarization assays, RAW264.7 macrophages were treated across three groups: Control, LPS (1 μg/mL, 24 h), and LPS + JBT (LPS + JBT 50 μg/mL, 24 h). For Western blot mechanistic studies, the same three treatment conditions were applied separately to normal, Atg5‐OE, and Atg5‐KD macrophages, generating nine groups in total.

### 2.6 Flow Cytometry

Apoptosis: Annexin V‐FITC/PI double staining; total apoptosis = early (Q4: Annexin V^+^/PI^−^) + late (Q2: Annexin V^+^/PI^+^). ΔΨm: JC‐1 reagent; red (aggregate)/green (monomer) fluorescence ratio. ROS: DCFH‐DA (10 μM, 20 min, 37°C); FITC channel fluorescence distribution. M1/M2 polarization: cells were stained with F4/80‐PE/Cy5, CD197‐FITC, and CD206‐APC after fixation and permeabilization. F4/80^+^ macrophages were gated first; CD197^+^ (M1) and CD206^+^ (M2) populations were then quantified within the F4/80^+^ gate; the bivariate plots shown in Figure 5a display quadrant percentages of total acquired events, whereas the M1 and M2 percentages summarized in Figure 5b–d were determined within the F4/80^+^ gate. FMO controls established gating boundaries; minimum 10,000 events acquired per sample.

### 2.7 Western Blot

Proteins were extracted in RIPA buffer with protease and phosphatase inhibitors. Equal loading (20 μg/lane), SDS‐PAGE, PVDF transfer, 5% milk/TBST block, primary antibody overnight at 4°C, HRP secondary (1 h, RT), ECL detection; GAPDH served as loading reference and all target signals were normalized to GAPDH. Each Western blot experiment was performed with five independent biological replicates; representative blots are shown and densitometric values represent mean ± SD of the five replicates. For the Atg5 genetic‐manipulation experiments (Figure 6), normal, Atg5‐OE and Atg5‐KD macrophages were processed as three separate blot panels; quantitative comparisons were therefore made between treatment conditions within each genetic background, and differences between genetic backgrounds are described in terms of the within‐panel change relative to the corresponding LPS condition. Primary antibodies (all from Abcam, Cambridge, UK): anti‐Bax (ab32503, rabbit, 1:1000), anti‐Bcl‐2 (ab196495, rabbit, 1:1000), anti‐LC3B (ab48394, rabbit, 1:1000), anti‐p62/SQSTM1 (ab109012, rabbit, 1:1000), anti‐ATG5 (ab108327, rabbit, 1:1000), anti‐p‐p38MAPK (Thr180/Tyr182) (ab47363, rabbit, 1:1000), anti‐p‐NF‐κB p65 (Ser536) (ab76302, rabbit, 1:1000), anti‐COL II (ab34712, rabbit, 1:200), anti‐MMP‐13 (ab39012, rabbit, 1:1000), anti‐GAPDH (ab8245, mouse, 1:10000). Secondary antibodies: HRP‐conjugated goat anti‐rabbit IgG (ab6721, 1:2000) and HRP‐conjugated goat anti‐mouse IgG (ab6789, 1:2000).

### 2.8 Immunofluorescence

NP cells: fixed 4% PFA (15 min), permeabilized 0.2% Triton X‐100, blocked 5% BSA; primary antibodies: Bax (red, Alexa Fluor 594), Bcl‐2 (green, Alexa Fluor 488), LC3 (far‐red, Alexa Fluor 647); Alexa Fluor secondaries (1 h, RT, dark); DAPI nuclear counterstain. Macrophages: same protocol using TNF‐α (purple), IL‐10 (red), LC3 (green). Images were acquired by fluorescence microscopy; representative fields are shown together with three‐dimensional fluorescence intensity surface plots generated in ImageJ. Immunofluorescence was used for spatial visualization and was not subjected to formal quantitative statistical analysis.

### 2.9 RT‐qPCR

Total RNA extracted (Animal Total RNA Kit); cDNA from 500 ng RNA (QuantiTect RT Kit, Qiagen); qPCR with SYBR Green Master Mix (Applied Biosystems); 2^−^^ΔΔCT^ method; GAPDH served as the internal reference gene. Primer sequences in Supplementary Table S1.

### 2.10 ELISA

TNF‐α, IL‐10, and IL‐1β quantified in rat serum, NP cell supernatants, and macrophage supernatants using commercial ELISA kits per manufacturer's instructions; absorbance at 450 nm; concentrations from four‐parameter logistic regression standard curves.

### 2.11 Statistical Analysis

Data are expressed as mean ± SD. No formal a priori power calculation was performed; the number of animals analyzed per endpoint was based on group sizes commonly used in published studies employing the same rat caudal annular puncture model, and the disc height index (DHI) was regarded as the principal in vivo outcome measure. For in vivo experiments, *n* = 5 animals per group were analyzed for each endpoint. For in vitro experiments, *n* = 5 independent experiments per group were analyzed, unless otherwise stated in the corresponding figure legend. For Western blot, representative blot images were selected to display the clearest band resolution and densitometric values represent the mean ± SD of all five replicates. Normality was assessed by the Shapiro–Wilk test; variance homogeneity by Levene's test. Multi‐group comparisons: one‐way ANOVA with Tukey's post hoc (normal/equal variance) or Kruskal‐Wallis with Dunn's correction (otherwise). All tests two‐tailed; *p* < 0.05 significant. SPSS 21.0 (IBM); graphs in GraphPad Prism; chromatographic fingerprint similarity was computed in Python 3.11 (NumPy/SciPy). Significance: *p < 0.05; **p < 0.01; ***p < 0.001; ns, not significant.

## 3 Results

### 3.1 HPLC Fingerprinting Confirms Reproducible Chemical Composition of Juanbi Tang

To characterize the JBT preparation and validate batch reproducibility, HPLC fingerprinting was performed with osthole as a quantitative reference standard (Figure 1). A linear standard curve for osthole was constructed across 20–160 μg/mL (*R* ^2^ > 0.999; Figure 1a), confirming quantitative reliability. The standard solution (160 μg/mL) yielded a single peak at 30.927 min with a theoretical plate number of 16,188 (USP), satisfying system suitability (Figure 1b). The inter‐batch RSD of osthole peak area across three independent preparations was 2.3% (Figure 1c), well below the 5% acceptance criterion mandated for quality control, confirming manufacturing reproducibility. The HPLC fingerprint of the JBT concentrated preparation displayed a complex multi‐peak profile consistent with the eleven‐herb polypharmacological composition (Figure 1d); the osthole peak was identified at 30.962 min by co‐elution with reference standard (arrow), and osthole content in the concentrated preparation was 91.90 μg/mL. This profile is qualitatively consistent with the 44‐constituent HPLC‐Q‐TOF characterization of JBT reported by Wang et al. [10].

To extend quality control beyond a single marker constituent, a multi‐component fingerprint similarity analysis was performed (Figure 1e). Sixteen common peaks were resolved between 8 and 43 min, with the osthole peak designated as the reference (S) peak. The relative peak area profiles of the decoction and of the vacuum‐concentrated preparation were closely superimposable, with a similarity of 0.9994 by the cosine angle method and 0.9989 by the correlation coefficient method; on replicate analysis, the RSD of relative peak area across all sixteen common peaks was below 3.3% (mean 0.86%). These data indicate that the multi‐constituent chromatographic profile of JBT is preserved through the concentration step and is reproducible on repeated analysis, and, together with the inter‐batch osthole RSD, provide two complementary lines of quality‐control evidence.

### 3.2 Juanbi Tang Preserves Disc Integrity Across Five Complementary Assessment Dimensions In Vivo

**Figure 2:**
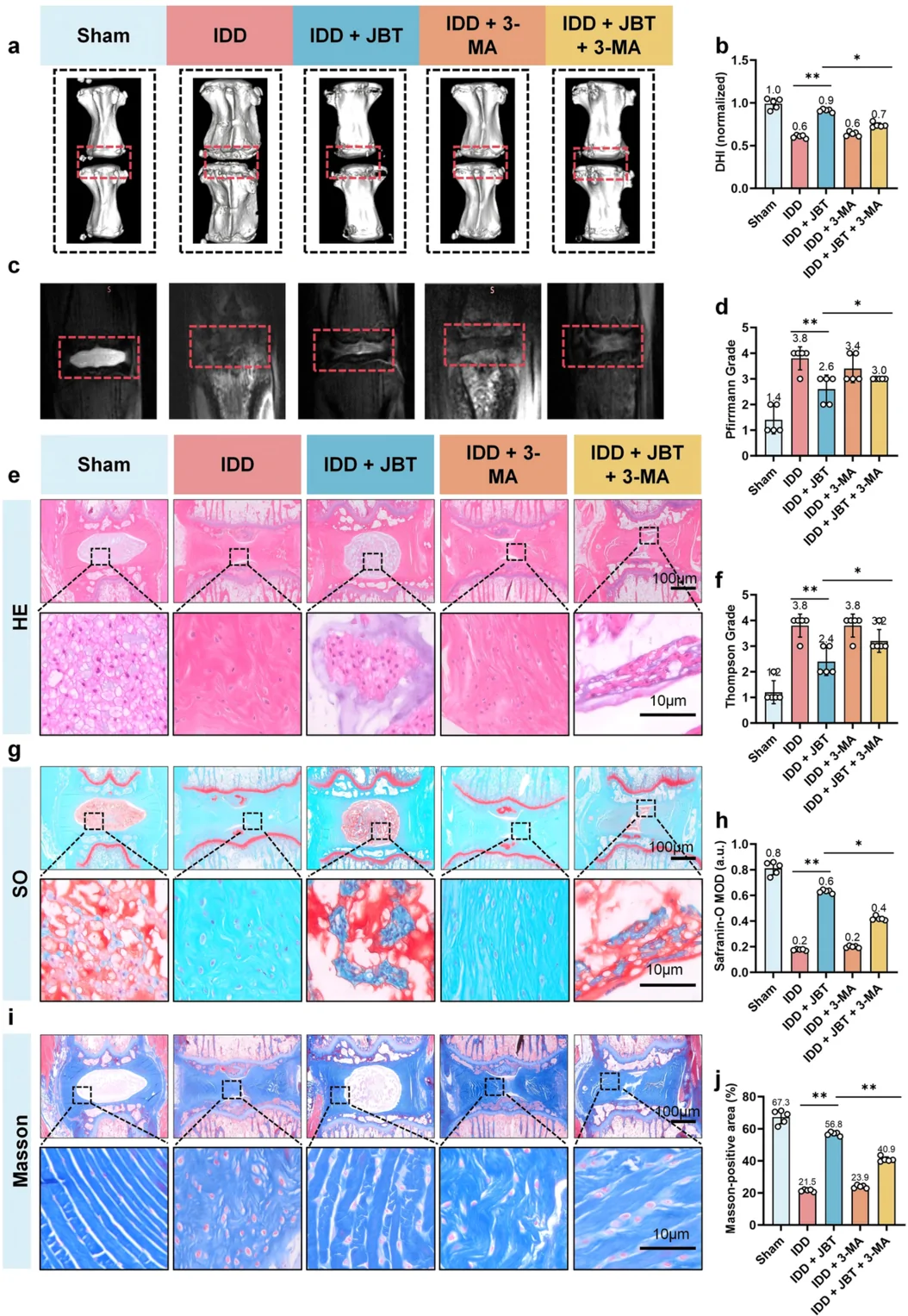
Juanbi Tang attenuates IDD across five assessment dimensions in vivo. Five groups: Sham, IDD, IDD + JBT, IDD + 3‐MA, IDD + JBT + 3‐MA. (a) Representative CT sagittal reconstructions. (b) DHI quantification (Sham 1.0, IDD 0.6, IDD + JBT 0.9, IDD + 3‐MA 0.6, IDD + JBT + 3‐MA 0.7). (c) Representative MRI T2‐weighted sagittal images. (d) Pfirrmann grade quantification (Sham 1.4, IDD 3.8, IDD + JBT 2.6, IDD + 3‐MA 3.4, IDD + JBT + 3‐MA 3.0). (e) Representative HE‐stained sections (scale bar: 100 μm, inset 10 μm). (f) Thompson grade quantification (Sham 1.2, IDD 3.8, IDD + JBT 2.4, IDD + 3‐MA 3.8, IDD + JBT + 3‐MA 3.2). (g) Representative Safranin O–Fast Green sections; red staining indicates proteoglycan‐rich NP matrix (scale bar: 100 μm, inset 10 μm). (h) SO MOD quantification (Sham 0.8, IDD 0.2, IDD + JBT 0.6, IDD + 3‐MA 0.2, IDD + JBT + 3‐MA 0.4). (i) Representative Masson trichrome sections; blue staining indicates collagen fibers (scale bar: 100 μm, inset 10 μm). (j) Masson‐positive area fraction (Sham 67.3%, IDD 21.5%, IDD + JBT 56.8%, IDD + 3‐MA 23.9%, IDD + JBT + 3‐MA 40.9%). Data are mean ± SD, *n* = 5 animals per group. *p < 0.05, **p < 0.01 by one‐way ANOVA with Tukey's post hoc; ns, not significant.

To evaluate JBT therapeutic efficacy in IDD, a rat annular puncture model was established and five complementary disc assessment modalities applied at 4 weeks post‐surgery: MRI, CT, SO staining, Masson trichrome, and HE staining (Figure 2). This multi‐modal strategy was designed to capture distinct biological dimensions of disc pathology — water content, structural height, proteoglycan composition, collagen organization, and cellular architecture — providing convergent evidence for disease modification.

CT imaging demonstrated progressive disc space narrowing in IDD animals (DHI: Sham 1.0 vs. IDD 0.6); JBT significantly preserved DHI (0.9; *p* < 0.01 vs. IDD; Figure 2a,b). MRI T2‐weighted imaging confirmed progressive NP signal loss in IDD animals; Pfirrmann grading was significantly lower in the IDD + JBT group relative to IDD controls (Sham 1.4 vs. IDD 3.8 vs. IDD + JBT 2.6; *p* < 0.01; Figure 2c,d). Partial reversal of CT and MRI benefits by 3‐MA co‐administration (IDD + JBT + 3‐MA: DHI 0.7, Pfirrmann 3.0; *p* < 0.05 vs. IDD + JBT) indicates autophagy dependence of JBT's structural protective activity.

HE staining confirmed NP cell vacuolization, nuclear condensation, and sparse cellular distribution in IDD discs, features substantially attenuated in JBT‐treated discs; Thompson grading confirmed significantly lower histological degeneration severity in the IDD + JBT group (Sham 1.2 vs. IDD 3.8 vs. IDD + JBT 2.4; *p* < 0.01 vs. IDD; Figure 2e,f). Safranin O–Fast Green staining revealed near‐complete proteoglycan depletion in IDD NP zones (MOD: Sham 0.8 vs. IDD 0.2); JBT‐treated discs maintained substantially higher proteoglycan content (MOD 0.6; *p* < 0.01 vs. IDD; Figure 2g,h). Masson trichrome staining revealed abundant, well‐organized blue‐stained collagen in Sham discs, which was extensively lost in IDD discs, reflecting the disruption and disorganization of the collagenous matrix that accompanies advanced disc degeneration; JBT largely preserved collagen content and fiber organization (Masson‐positive area: Sham 67.3%, IDD 21.5%, IDD + JBT 56.8%; *p* < 0.01 vs. IDD; Figure 2i,j). Consistent autophagy dependence (3‐MA partial reversal across all five endpoints) was confirmed: IDD + JBT + 3‐MA showed intermediate values for Thompson grade (3.2), SO MOD (0.4), Pfirrmann grade (3.0), and Masson‐positive area (40.9%) relative to the IDD + JBT and IDD groups.

### 3.3 Juanbi Tang Suppresses the p38MAPK/NF‐κB Pathway and Coordinates Local and Systemic Anti‐Inflammatory Responses In Vivo

**Figure 3:**
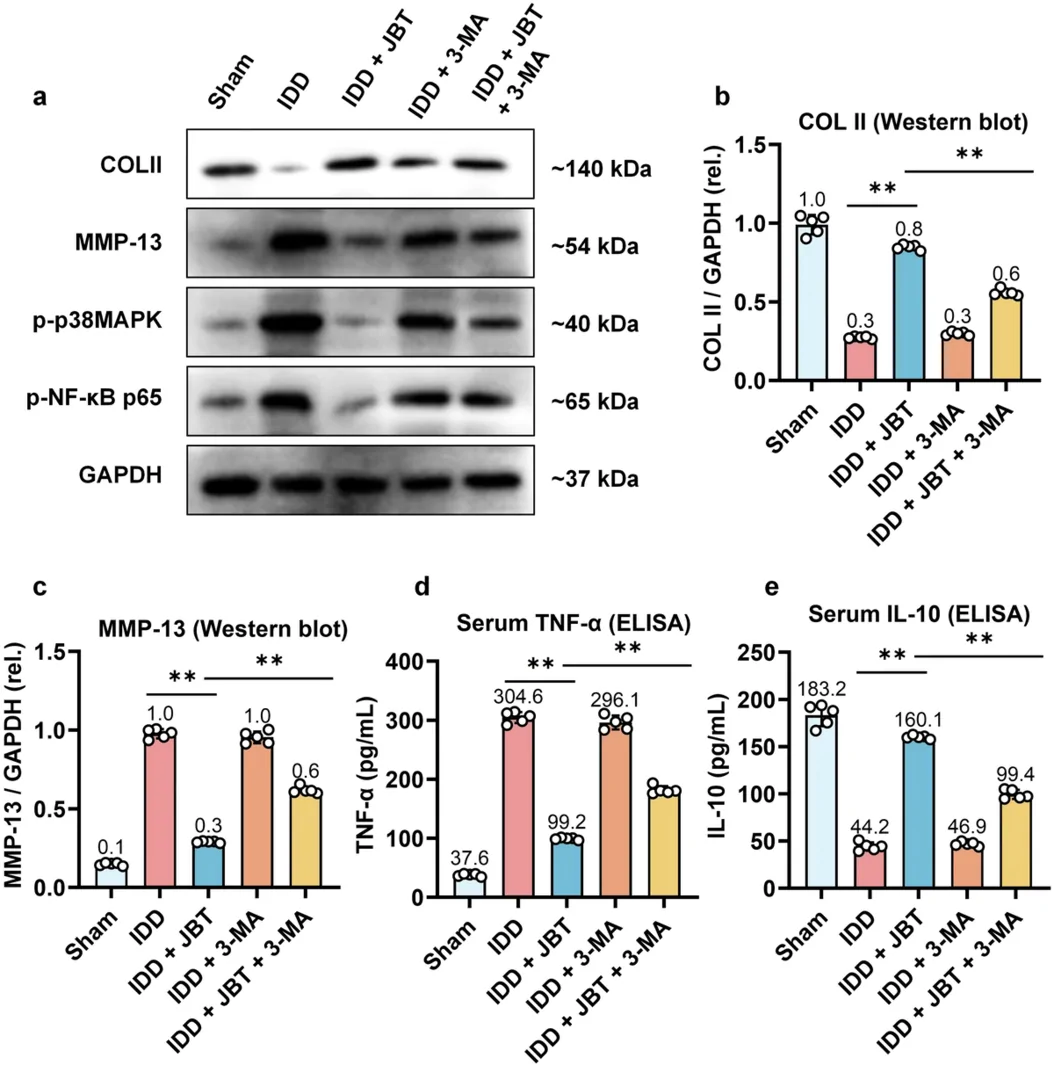
Juanbi Tang suppresses p38MAPK/NF‐κB signaling and modulates the inflammatory cytokine environment in vivo. (a) Western blot of disc tissue for COL II (∼140 kDa), MMP‐13 (∼54 kDa), p‐p38MAPK (∼40 kDa), p‐NF‐κB p65 (∼65 kDa), and GAPDH (∼37 kDa) across five groups (Sham, IDD, IDD + JBT, IDD + 3‐MA, IDD + JBT + 3‐MA); all targets were normalized to GAPDH. (b) COL II/GAPDH densitometry (Sham 1.0, IDD 0.3, IDD + JBT 0.8, IDD + 3‐MA 0.3, IDD + JBT + 3‐MA 0.6). (c) MMP‐13/GAPDH densitometry (Sham 0.1, IDD 1.0, IDD + JBT 0.3, IDD + 3‐MA 1.0, IDD + JBT + 3‐MA 0.6). (d) Serum TNF‐α by ELISA (pg/mL; Sham 37.6, IDD 304.6, IDD + JBT 99.2, IDD + 3‐MA 296.1, IDD + JBT + 3‐MA 169.1). (e) Serum IL‐10 by ELISA (pg/mL; Sham 183.2, IDD 44.2, IDD + JBT 160.1, IDD + 3‐MA 46.9, IDD + JBT + 3‐MA 99.4). Data are mean ± SD, *n* = 5 animals per group. *p < 0.05, **p < 0.01 by one‐way ANOVA with Tukey's post hoc; ns, not significant.

To elucidate the molecular basis of JBT's disease‐modifying activity, Western blot and ELISA analyses were performed on disc tissue and serum across all five experimental groups (Figure 3).

Western blot of disc tissue demonstrated selective elevation of p‐p38MAPK and p‐NF‐κB p65 in IDD animals, confirming pathway‐specific activation (Figure 3a). JBT significantly reduced both p‐p38MAPK/GAPDH and p‐NF‐κB p65/GAPDH relative to IDD controls (*p* < 0.01; Figure 3a). COL II was markedly depleted in IDD disc tissue (COL II/GAPDH: Sham 1.0 vs. IDD 0.3) and substantially restored by JBT (0.8; *p* < 0.01 vs. IDD; Figure 3b). MMP‐13 was markedly elevated in IDD tissue (MMP‐13/GAPDH: Sham 0.1 vs. IDD 1.0) and significantly suppressed by JBT (0.3; *p* < 0.01 vs. IDD; Figure 3c), shifting the disc matrix from net‐catabolism toward net‐preservation. 3‐MA co‐treatment partially reversed these molecular changes (IDD + JBT + 3‐MA: COL II/GAPDH 0.6, MMP‐13/GAPDH 0.6; both *p* < 0.01 vs. IDD + JBT; Figure 3b,c).

ELISA demonstrated markedly elevated serum TNF‐α in IDD animals (Sham 37.6 vs. IDD 304.6 pg/mL), substantially reduced by JBT to 99.2 pg/mL (*p* < 0.01 vs. IDD; Figure 3d). Conversely, serum IL‐10 was significantly depleted in IDD animals (Sham 183.2 vs. IDD 44.2 pg/mL) and substantially restored by JBT to 160.1 pg/mL (*p* < 0.01 vs. IDD; Figure 3e). These systemic cytokine changes mirror the local disc molecular findings, indicating that JBT coordinates immunomodulation at both tissue and systemic levels. 3‐MA co‐treatment partially attenuated JBT‐mediated cytokine modulation (IDD + JBT + 3‐MA: TNF‐α 169.1 pg/mL, IL‐10 99.4 pg/mL), further corroborating autophagy dependence in vivo.

### 3.4 Juanbi Tang Protects Nucleus Pulposus Cells Through Concurrent Anti‐Apoptotic, Mitochondrial‐Protective, and Autophagy‐Activating Mechanisms

**Figure 4:**
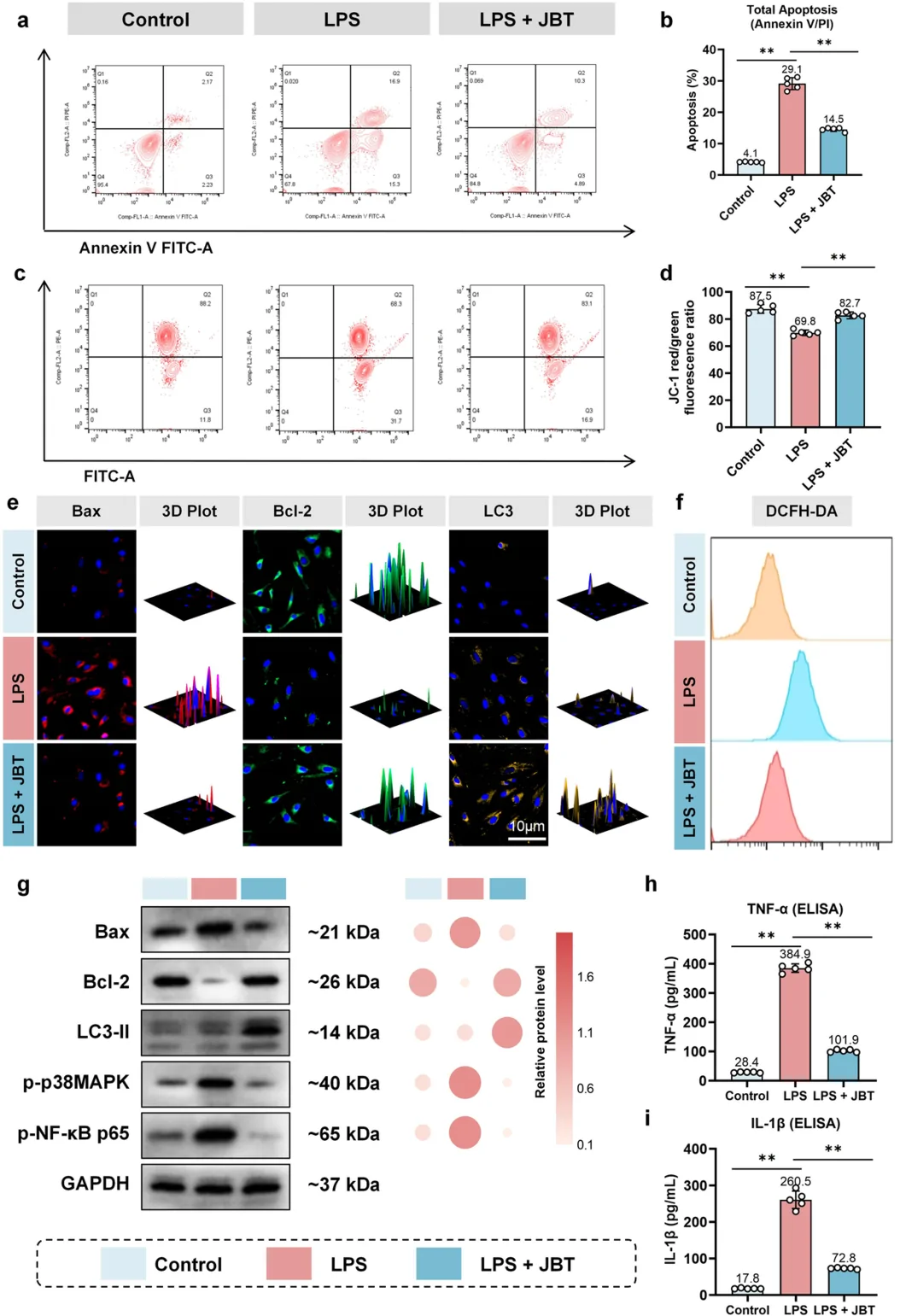
Juanbi Tang provides multi‐dimensional cytoprotection to NP cells. Three groups: Control, LPS (1 μg/mL, 24 h), LPS + JBT (50 μg/mL, 24 h). (a) Annexin V‐FITC/PI scatter plots. (b) Total apoptosis rate quantification (Control 4.1%, LPS 29.1%, LPS + JBT 14.5%). (c) JC‐1 scatter plots for mitochondrial membrane potential (ΔΨm). (d) JC‐1 red/green fluorescence ratio (Control 87.5, LPS 69.8, LPS + JBT 82.7). (e) Immunofluorescence: Bax (red, Alexa Fluor 594), Bcl‐2 (green, Alexa Fluor 488), LC3 (far‐red, Alexa Fluor 647), DAPI (blue); scale bar 10 μm; three‐dimensional fluorescence intensity plots shown alongside each channel; images are representative and were not subjected to formal quantitative statistical analysis. (f) DCFH‐DA overlaid histograms (FITC channel) for intracellular ROS. (g) Western blot — Bax (∼21 kDa), Bcl‐2 (∼26 kDa), LC3B (LC3‐II, ∼14 kDa), p‐p38MAPK (∼40 kDa), p‐NF‐κB p65 (∼65 kDa), GAPDH (∼37 kDa); bubble chart represents relative protein level normalized to GAPDH. (h) TNF‐α ELISA (Control 28.4, LPS 384.9, LPS + JBT 101.9 pg/mL). (i) IL‐1β ELISA (Control 17.8, LPS 260.5, LPS + JBT 72.8 pg/mL). Data are mean ± SD, *n* = 5 independent experiments per group. *p < 0.05, **p < 0.01 by one‐way ANOVA with Tukey's post hoc; ns, not significant.

Primary rat NP cells were subjected to LPS (1 μg/mL) injury with or without JBT (50 μg/mL) across three groups—Control, LPS (1 μg/mL, 24 h), and LPS + JBT (50 μg/mL, 24 h)—to simultaneously characterize cytoprotective and mechanistic effects (Figure 4).

Annexin V/PI flow cytometry showed that LPS markedly induced NP cell apoptosis (Control: 4.1%; LPS: 29.1%; *p* < 0.01; Figure 4a,b). JBT reduced total apoptosis to 14.5% (*p* < 0.01 vs. LPS), indicating upstream intervention in the apoptotic cascade.

ΔΨm assessed by JC‐1 revealed mitochondrial depolarization in LPS‐treated NP cells (JC‐1 red/green fluorescence ratio: Control 87.5 vs. LPS 69.8; *p* < 0.01; Figure 4c,d); JBT significantly restored ΔΨm (82.7; *p* < 0.01 vs. LPS). DCFH‐DA flow cytometric analysis confirmed that JBT attenuated LPS‐induced ROS accumulation, as shown by the leftward shift of the fluorescence histogram relative to the LPS group (Figure 4f).

Immunofluorescence staining provided spatially resolved confirmation (Figure 4e). LPS‐treated NP cells exhibited markedly elevated diffuse Bax (red) with perinuclear condensation, near‐absent Bcl‐2 (green), and sparse LC3 puncta (far‐red, Alexa Fluor 647), with nuclear condensation visible by DAPI. JBT treatment substantially reversed all three patterns: Bax fluorescence diminished, Bcl‐2 was robustly restored, and LC3 puncta became densely distributed throughout the cytoplasm, indicating active autophagosome formation.

Western blot confirmed LPS‐induced Bax elevation and Bcl‐2 suppression; JBT significantly reversed the Bax/Bcl‐2 ratio (*p* < 0.01 vs. LPS; Figure 4g). JBT increased LC3‐II and decreased p62/SQSTM1, indicating autophagy flux activation, while concurrently suppressing p‐p38MAPK and p‐NF‐κB p65, recapitulating in NP cells the pathway‐selective inhibition observed in disc tissue in vivo. ELISA of NP cell supernatants confirmed significant JBT‐mediated suppression of LPS‐induced TNF‐α (384.9 → 101.9 pg/mL; *p* < 0.01; Figure 4h) and IL‐1β secretion (260.5 → 72.8 pg/mL; *p* < 0.01; Figure 4i). RT‐qPCR analysis of the same targets showed concordant changes at the transcript level (Supplementary Figure S1).

### 3.5 Juanbi Tang Promotes Macrophage M2 Polarization In Vitro

**Figure 5:**
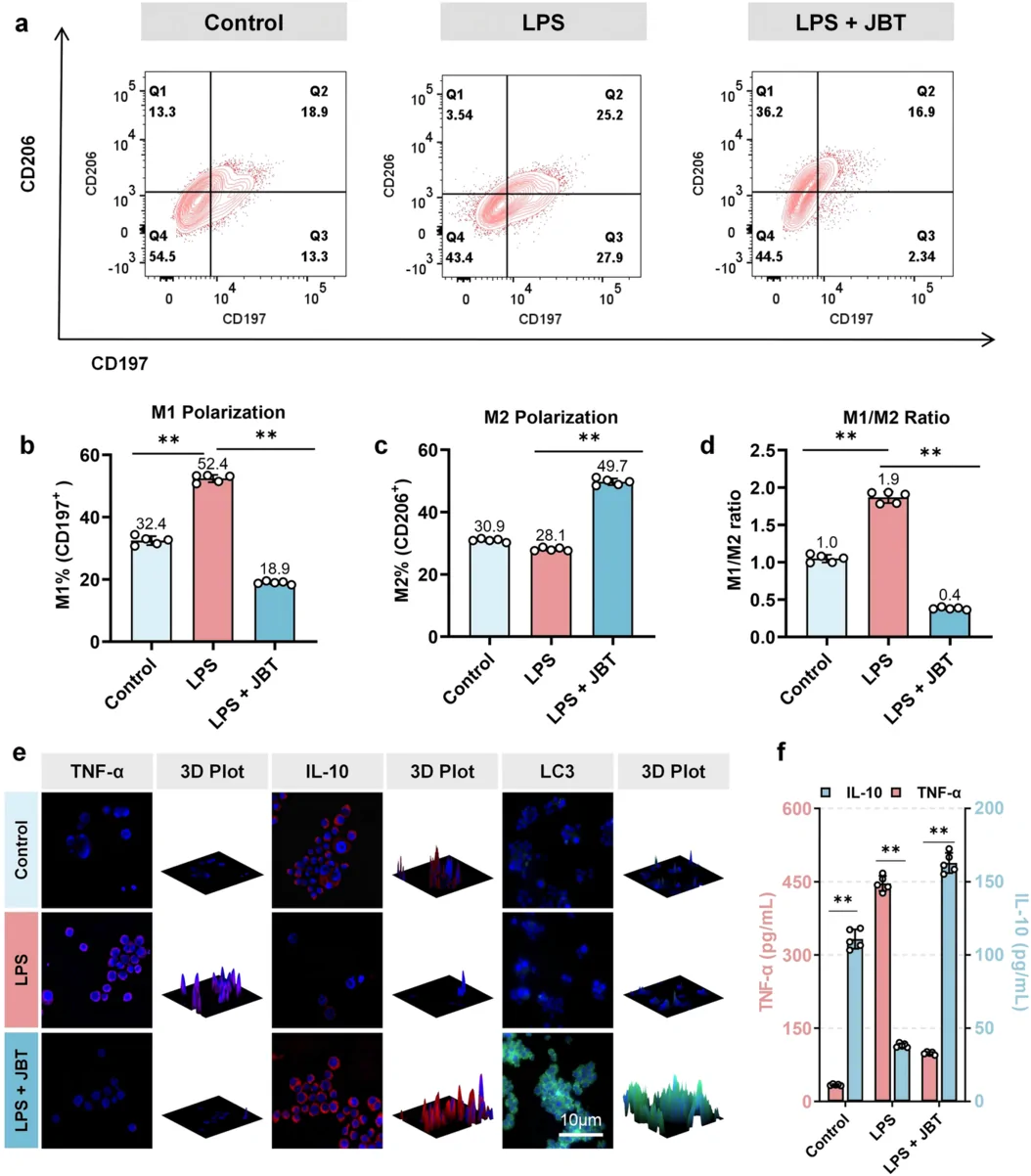
Juanbi Tang promotes macrophage M2 polarization in vitro. Three groups: Control, LPS (1 μg/mL, 24 h), LPS + JBT (50 μg/mL, 24 h). (a) Flow cytometry bivariate plots (x‐axis: CD197; y‐axis: CD206) (Control: Q1 13.3%/Q2 18.9%/Q3 13.3%/Q4 54.5%; LPS: Q1 3.54%/Q2 25.2%/Q3 27.9%/Q4 43.4%; LPS + JBT: Q1 36.2%/Q2 16.9%/Q3 2.34%/Q4 44.5%). Quadrant percentages in (a) refer to total acquired events, whereas the values in (b–d) were determined within the F4/80^+^ gate; the two are therefore not numerically identical. (b) M1 polarization (CD197^+^, %): Control 32.4%, LPS 52.4%, LPS + JBT 18.9%. (c) M2 polarization (CD206^+^, %): Control 30.9%, LPS 28.1%, LPS + JBT 49.7%. (d) M1/M2 ratio: Control 1.0, LPS 1.9, LPS + JBT 0.4. (e) Immunofluorescence: TNF‐α (purple), IL‐10 (red), LC3 (green); scale bar 10 μm; three‐dimensional fluorescence intensity plots shown alongside; images are representative and were not subjected to formal quantitative statistical analysis. (f) ELISA: TNF‐α (left axis, pg/mL) and IL‐10 (right axis, pg/mL) in macrophage supernatants. Data are mean ± SD, *n* = 5 independent experiments per group. *p < 0.05, **p < 0.01 by one‐way ANOVA with Tukey's post hoc; ns, not significant.

To investigate whether JBT modulates macrophage polarization, murine RAW264.7 macrophages were allocated to three groups—Control, LPS (1 μg/mL), and LPS + JBT (50 μg/mL)—and polarization state was assessed by flow cytometry, immunofluorescence, and ELISA (Figure 5).

Flow cytometric quantification of CD197^+^ (M1) and CD206^+^ (M2) populations revealed distinct polarization patterns across the three groups (Figure 5a–d). Control macrophages exhibited an M1/M2 ratio of approximately 1.0, with CD197^+^ (M1) cells at 32.4% and CD206^+^ (M2) cells at 30.9%. LPS stimulation markedly shifted the balance toward M1 polarization: CD197^+^ cells increased to 52.4% while CD206^+^ cells decreased to 28.1%, yielding a significantly elevated M1/M2 ratio of 1.9 (*p* < 0.01 vs. Control; Figure 5d). JBT treatment strongly reversed this M1 shift: CD197^+^ cells decreased to 18.9% (*p* < 0.01 vs. LPS; Figure 5b), while CD206^+^ cells markedly increased to 49.7% (*p* < 0.01 vs. LPS; Figure 5c), reducing the M1/M2 ratio to 0.4 (*p* < 0.05 vs. LPS). These data demonstrate that JBT significantly promotes M2 polarization in LPS‐challenged macrophages, consistent with classical M1‐to‐M2 reprogramming.

Immunofluorescence staining for TNF‐α (purple), IL‐10 (red), and LC3 (green) was consistent with the polarization findings at single‐cell resolution (Figure 5e). LPS‐stimulated macrophages exhibited prominently elevated purple TNF‐α fluorescence and diminished red IL‐10 signal. JBT‐treated macrophages showed markedly reduced TNF‐α (purple), elevated IL‐10 (red), and increased green LC3 puncta density, indicating concurrent M2 promotion and autophagy activation.

ELISA quantification of macrophage supernatants confirmed that JBT significantly suppressed LPS‐induced TNF‐α secretion and elevated IL‐10 secretion relative to the LPS‐only group (both *p* < 0.01; Figure 5f), providing functional validation of the M2 polarization shift at the cytokine secretion level.

### 3.6 Atg5 Expression Level Determines the Degree of p38MAPK/NF‐κB Suppression in Macrophages

**Figure 6:**
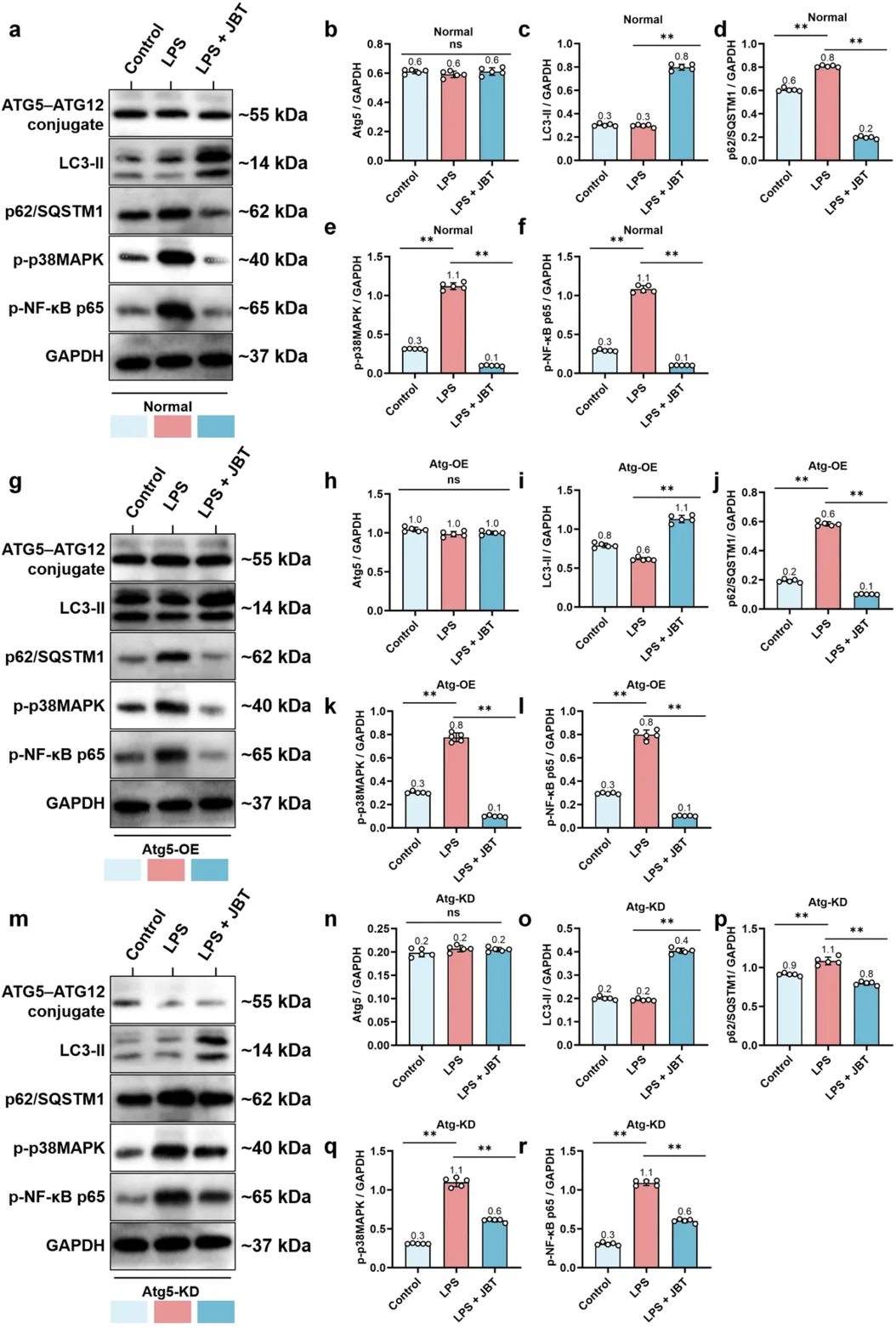
Atg5 expression level modulates the degree of p38MAPK/NF‐κB suppression by JBT in macrophages. Nine experimental groups: normal, Atg5‐OE, and Atg5‐KD RAW264.7 macrophages × Control/LPS (1 μg/mL)/LPS + JBT (50 μg/mL); presented as three separate blot panels. Western blot targets: ATG5–ATG12 conjugate (∼55 kDa), LC3B (LC3‐II, ∼14 kDa), p62/SQSTM1 (∼62 kDa), p‐p38MAPK (∼40 kDa), p‐NF‐κB p65 (∼65 kDa), GAPDH (∼37 kDa); all targets normalized to GAPDH. (a) Representative blots, normal macrophages. (b–f) Corresponding densitometry for ATG5–ATG12 conjugate, LC3‐II, p62/SQSTM1, p‐p38MAPK and p‐NF‐κB p65, respectively. (g) Representative blots, Atg5‐OE macrophages. (h–l) Corresponding densitometry for the same five targets. (m) Representative blots, Atg5‐KD macrophages. (n–r) Corresponding densitometry for the same five targets. Because the three genetic backgrounds were analyzed on separate membranes, statistical comparisons were made between treatment conditions within each panel. Color‐coded group labels: light blue = Control, salmon = LPS, teal = LPS + JBT. Data are mean ± SD, *n* = 5 independent experiments per group. **p < 0.01; ns, not significant by one‐way ANOVA with Tukey's post hoc.

To elucidate the molecular mechanism linking autophagy to sustained NF‐κB inhibition in macrophages, Atg5 was overexpressed (Atg5‐OE) or knocked down (Atg5‐KD) in RAW264.7 macrophages, and Western blot analysis was performed across all nine experimental groups (normal/OE/KD × Control/LPS/LPS + JBT) (Figure 6). Successful genetic manipulation was confirmed by Western blot: the ATG5–ATG12 conjugate was clearly more abundant in Atg5‐OE macrophages and clearly less abundant in Atg5‐KD macrophages than in normal macrophages (Figure 6a,g,m), and was stable across the three treatment conditions within each genetic background (ns; Figure 6b,h,n).

In normal macrophages, JBT treatment significantly increased LC3‐II (LPS 0.3 vs. JBT 0.8) and decreased p62/SQSTM1 (LPS 0.8 vs. JBT 0.2), while markedly suppressing p‐p38MAPK and p‐NF‐κB p65 (both: LPS 1.1 vs. JBT 0.1; all *p* < 0.01; Figure 6a–f), confirming pathway‐selective inhibition and concurrent autophagy activation. In Atg5‐OE macrophages, augmented autophagic capacity was accompanied by further LC3‐II accumulation (LPS 0.6 vs. JBT 1.1) and near‐complete p62 clearance (LPS 0.6 vs. JBT 0.1), with p‐p38MAPK and p‐NF‐κB p65 both reduced to 0.1 by JBT (all *p* < 0.01; Figure 6g–l).

In Atg5‐KD macrophages, autophagic capacity was severely blunted: LC3‐II accumulation was markedly impaired even with JBT treatment (LPS 0.2 vs. JBT 0.4), while p62/SQSTM1 remained elevated (LPS 1.1 vs. JBT 0.8), and suppression of p‐p38MAPK and p‐NF‐κB p65 by JBT was incomplete (both: LPS 1.1 vs. JBT 0.6; *p* < 0.01; Figure 6m–r). Because the three genetic backgrounds were analyzed on separate membranes, the effect of Atg5 level is most appropriately expressed as the within‐panel change produced by JBT relative to the corresponding LPS condition. On this basis, JBT reduced p62/SQSTM1 by approximately 75% in normal macrophages and 83% in Atg5‐OE macrophages, but by only approximately 27% in Atg5‐KD macrophages; p‐p38MAPK and p‐NF‐κB p65 were each reduced by approximately 91% and 88% in normal and Atg5‐OE macrophages, respectively, but by only approximately 45% in Atg5‐KD macrophages. Collectively, these data from three genetic contexts are consistent with a regulatory module: JBT suppresses p38MAPK/NF‐κB and activates Atg5‐dependent autophagy; autophagic p62 clearance consolidates NF‐κB inhibition; and the amplitude of this module is bidirectionally determined by Atg5 expression level. RT‐qPCR analysis of the same targets showed concordant changes at the transcript level (Supplementary Fig. S2).

## 4 Discussion

The present study provides systematic mechanistic characterization of Juanbi Tang's therapeutic activity in IDD, addressing a long‐standing gap between the formula's empirical clinical application in Bi syndrome—documented in Yi Xue Xin Wu since 1732—and the molecular understanding required for rational clinical translation. Our findings support a mechanistic model in which JBT concurrently suppresses the p38MAPK/NF‐κB signaling pathway and activates Atg5‐dependent autophagy. To avoid ambiguity, we use the term regulatory module throughout, in preference to “axis”, and we emphasize that the proposed cascade should be read as a mechanistic model rather than as an experimentally resolved linear causal chain: NF‐κB suppression reduces transcriptional drive on the p62/SQSTM1 gene (whose promoter contains functional NF‐κB response elements) [20], autophagic flux further degrades accumulated p62, and reduced p62 in turn consolidates NF‐κB inhibition by attenuating TRAF6–IKK scaffold activity [9, 21]. This autophagy–p62–NF‐κB regulatory module is consistent with mechanisms independently characterized in other inflammatory contexts [8, 20], and the Atg5 gain‐ and loss‐of‐function data presented here provide bidirectional support for its operation in macrophages. The resulting anti‐inflammatory phenotypic shift is associated with macrophage M2 polarization, attenuation of NP cell apoptosis, and preservation of disc structural integrity across multiple assessment dimensions [4].

A question raised directly by these data concerns the hierarchical relationship between p38MAPK inhibition and autophagy activation: whether the two are strictly parallel consequences of JBT treatment, or whether one lies upstream of the other. Our experiments were not designed to resolve this, but the published literature favors at least a partial upstream–downstream relationship. p38 has been shown to phosphorylate ATG5 at Thr75 through the Gadd45β–MEKK4 pathway, thereby inhibiting starvation‐induced autophagy, with autophagic flux correspondingly enhanced in p38‐deficient cells [22]; independently, p38α negatively regulates both basal and starvation‐induced autophagy by controlling mAtg9 trafficking through its interacting protein p38IP [23]. If either mechanism operates in disc‐infiltrating macrophages, JBT‐mediated suppression of p‐p38MAPK would be expected to relieve an inhibitory constraint on ATG5‐dependent autophagosome formation, placing p38MAPK inhibition upstream of, rather than merely concurrent with, autophagy activation. We did not measure ATG5 Thr75 phosphorylation, nor did we compare the autophagic phenotype produced by JBT with that produced by a selective p38 inhibitor; this hierarchical model therefore remains a hypothesis. Direct tests—in particular, determining whether SB203580 alone phenocopies the LC3‐II and p62 changes produced by JBT, and quantifying ATG5 Thr75 phosphorylation—are defined objectives of our ongoing work.

The five‐modality in vivo assessment strategy—MRI, CT, Safranin O, Masson trichrome, and HE staining—was intentionally designed to capture IDD pathology across distinct biological dimensions. The addition of Masson trichrome to the histological panel is a methodological strength: while Safranin O reflects proteoglycan content, Masson staining reveals collagen fiber organization and the integrity of the collagenous matrix, which is particularly relevant for assessing annular preservation. The convergence of significant JBT‐mediated protection across all five endpoints, and their consistent partial reversal by 3‐MA, provides multi‐layered evidence that autophagy contributes to disease modification across biological scales simultaneously.

JBT's polypharmacological chemical composition, confirmed by HPLC fingerprinting consistent with the 44‐constituent profile reported by Wang et al. [10], provides a molecular basis for its multi‐target activity. Osthole, from Angelica pubescens Maxim. f. biserrata Shan et Yuan, has documented p38MAPK‐suppressing activity in musculoskeletal tissues [12, 24, 25]; boswellic acids from Boswellia sacra Flueck. are established NF‐κB inhibitors [11, 26]; ferulic acid from Angelica sinensis (Oliv.) Diels suppresses MMP‐13 [27, 28]; gentiopicroside from Gentiana macrophylla Pall. inhibits p38/ERK/JNK‐mediated signaling [29, 30]. The availability of these pharmacophores within a single preparation explains the simultaneous pathway suppression, autophagy activation, and matrix restoration observed, consistent with the polypharmacological rationale encoded in the jun‐chen‐zuo‐shi compositional hierarchy.

The mechanistic role of autophagy in this study is supported by two independent experimental approaches—3‐MA pharmacological inhibition in vivo, and Atg5 genetic gain‐ and loss‐of‐function in vitro—each providing complementary evidence from different experimental contexts. The 3‐MA data indicate that autophagy contributes to JBT's disease‐modifying effects in the intact animal [31, 32]; while 3‐MA has recognized non‐specificity as a PI3K inhibitor, its consistent partial reversal of JBT‐mediated benefits across all five structural and molecular endpoints in vivo provides coherent evidence of an autophagy‐dependent component. Future studies employing more selective pharmacological tools, such as Beclin‐1 knockdown or VPS34 catalytic‐domain inhibitors, or conditional autophagy‐knockout animal models, would provide more definitive mechanistic attribution independent of PI3K class effects. The Atg5 Western blot data provide a more specific molecular dissection, demonstrating that Atg5 expression level bidirectionally modulates the degree of p62 clearance and NF‐κB suppression achievable by JBT.

The macrophage polarization data demonstrate that JBT significantly promotes M2 polarization in LPS‐stimulated RAW264.7 macrophages, reducing the M1/M2 ratio and elevating IL‐10 secretion. This polarization shift, combined with concurrent LC3 puncta accumulation observed by immunofluorescence, is consistent with the mechanistic model in which pathway suppression and autophagy activation together facilitate M2 phenotype adoption. An important consideration is the species mismatch between the in vitro (murine RAW264.7) and in vivo (rat) systems. RAW264.7 cells were selected as a widely used and genetically tractable macrophage system suitable for Atg5 gain‐ and loss‐of‐function dissection, and the p38MAPK/NF‐κB and Atg5‐dependent autophagy pathways examined here are highly conserved between mouse and rat, with extensive published evidence of functionally equivalent responses to LPS stimulation and autophagy modulation in both species. Nevertheless, the macrophage findings should be interpreted with appropriate caution, and they are best regarded as a mechanistic dissection of a conserved pathway rather than as a direct cross‐species extrapolation of efficacy. Confirmation of the key polarization and autophagy findings in rat primary peritoneal or bone marrow‐derived macrophages, or in a rat macrophage line, is a priority for our subsequent work. The direct functional link between JBT‐induced M2 polarization and NP cell protection — while demonstrated here in separate experimental systems — is consistent with prior co‐culture and conditioned medium studies in which M2‐polarized macrophage secretomes have been shown to attenuate NP cell apoptosis and restore ECM homeostasis [4, 5]. Future studies employing Transwell co‐culture of JBT‐conditioned macrophages with rat primary NP cells would directly confirm this paracrine mechanism.

The comprehensive NP cell protection documented in Section 3.4 bridges the immunological mechanism to the cellular outcome directly relevant to disc biology. JBT's simultaneous suppression of apoptosis, restoration of ΔΨm, attenuation of ROS, and activation of autophagy across the same NP cell system indicates that these mitochondrial parameters are mechanistically coordinated — consistent with the role of mitochondrial outer membrane permeabilization in orchestrating intrinsic apoptosis. The clinical significance of mitochondrial protection in IDD is underscored by the disc's avascular, nutrient‐limited microenvironment, which renders NP cells chronically dependent on mitochondrial efficiency and uniquely vulnerable to bioenergetic collapse [33].

### 4.1 Limitations

Several limitations should be acknowledged explicitly. First, the study did not include a clinical positive control drug such as celecoxib or rapamycin. The absence of such a comparator means that, although JBT produced consistent and statistically significant benefits across five independent in vivo endpoints, the magnitude of its effect cannot be benchmarked directly against established anti‐inflammatory or autophagy‐inducing agents, and the clinical reference value of the efficacy data is correspondingly limited. A head‐to‐head comparison against celecoxib and rapamycin, in both the rat annular puncture model and the macrophage system, is planned as the next stage of this work.

Second, as discussed above, the in vitro macrophage work employed the murine RAW264.7 line whereas the in vivo model was performed in rats; cross‐species inference is therefore constrained and the macrophage data should be read as mechanistic rather than as species‐matched efficacy evidence.

Third, quality control combined single‐marker quantification of osthole with multi‐component fingerprint similarity analysis; simultaneous quantification of multiple constituents by UPLC‐MS/MS is planned for subsequent investigation.

Fourth, the in vitro studies employed direct application of JBT aqueous extract, a widely adopted approach for TCM mechanistic research that permits controlled dose delivery; the concentration used (50 μg/mL) was confirmed as the maximum sub‐toxic effective concentration by preliminary CCK‐8 assay, and studies using medicated serum are planned.

Fifth, Transwell co‐culture of JBT‐conditioned macrophages with primary NP cells is planned to establish the paracrine link between M2 polarization and NP cell protection.

#### 4.1.1 Conclusion

In summary, Juanbi Tang, a classical Chinese herbal formula with nearly 300 years of clinical application in musculoskeletal Bi syndrome, attenuates IDD, at least in part, through a regulatory cascade consistent with a coherent mechanistic model. JBT suppresses the p38MAPK/NF‐κB pathway, activates Atg5‐dependent autophagy, and promotes macrophage M1‐to‐M2 polarization through an autophagy–p62–NF‐κB regulatory module, which is associated with attenuation of NP cell apoptosis and preservation of disc structural integrity across five assessment dimensions. The Atg5 gain‐ and loss‐of‐function data indicate that autophagic p62 clearance functions as a key modulator of JBT's primary pharmacological signal. HPLC fingerprinting and multi‐component similarity analysis reveal the polypharmacological chemical complexity of the preparation, consistent with published multi‐constituent characterization [10]. These findings offer mechanistic support for the traditional use of JBT in degenerative spinal conditions and suggest a framework for its rational evaluation in clinical IDD management.

## Author Contributions

**Ye‐Hui Wang:** conceptualization, methodology, investigation, data curation, writing – original draft. **Wei Hou:** investigation, data curation, formal analysis. **Guo‐Sheng Tang:** investigation, formal analysis. **Xuan‐Geng Deng:** investigation, resources. **Si‐Mao Song:** investigation. **Wei Cui:** investigation. **Yang Ye:** visualization, data curation, software. **Yang Yu:** conceptualization, supervision, project administration.

## AI Usage Declaration

During the preparation of this manuscript, the authors used an artificial intelligence tool for language editing and for refining the wording of the Discussion. All data analysis, interpretation and scientific conclusions are the authors’ own. All AI‐assisted text was carefully reviewed, verified, and edited by the authors, who take full responsibility for the accuracy and integrity of the final manuscript. No artificial intelligence tool was used for the generation, analysis, or interpretation of experimental data, or for the creation or manipulation of images.

## Ethics Statement

All animal procedures were reviewed and approved by the Animal Ethics Committee of West China Hospital, Sichuan University (approval No. 20231116001, 16 November 2023). Experimental protocols were designed and executed in full compliance with the internationally accepted principles for laboratory animal use and care, including the European Community guidelines (EEC Directive of 1986; 86/609/EEC) and the US guidelines (NIH publication #85‐23, revised 1985), as well as the national standards for the humane care and use of laboratory animals (China, GB/T 35892‐2018).

## Conflicts of Interest

The authors declare no conflicts of interest.

## Supporting information


Supporting File:

## Data Availability

All data generated or analysed during this study are included in the published article and its supplementary materials. Primary numerical data (flow cytometry values, ELISA concentrations, Western blot densitometry) are presented in the figures. Raw data are available from the corresponding author upon reasonable request.

## References

[1] GBD 2021 Low Back Pain Collaborators . Global, Regional, and National Burden of Low Back Pain, 1990‐2020, its Attributable Risk Factors, and Projections to 2050,” Lancet Rheumatology 5 (2023): e316–e329. 37273833 10.1016/S2665-9913(23)00098-X PMC10234592

[2] N. N. Knezevic , K. D. Candido , J. W. S. Vlaeyen , J. Van Zundert , and S. P. Cohen , “Low Back Pain,” Lancet 398 (2021): 78–92. 34115979 10.1016/S0140-6736(21)00733-9

[3] J. Koroth , E. O. Buko , R. Abbott , et al., “Macrophages and Intervertebral Disc Degeneration,” International Journal of Molecular Sciences 24 (2023): 1367. 36674887 10.3390/ijms24021367 PMC9863885

[4] Y. Zhao , H. Tian , Y. Zhu , et al., “Role of Macrophage in Intervertebral Disc Degeneration,” Bone Research 13 (2025): 8. 39848963 10.1038/s41413-024-00397-7 PMC11758090

[5] Z. Hu , Y. Ma , Z. Wang , et al., “Mechanisms and Therapeutic Strategies of Macrophage Polarization in Intervertebral Disc Degeneration,” Journal of Orthopaedic Translation 52 (2025): 27–41.40231159

[6] G. Z. Zhang , M. Q. Liu , H. W. Chen , et al., “NF‐κB Signalling Pathways in Nucleus Pulposus Cell Function and Intervertebral Disc Degeneration,” Cell Proliferation 54 (2021): e13057. 34028920 10.1111/cpr.13057 PMC8249791

[7] H. Zhang , P. Song , Y. Tang , X. L. Zhang , Y. Shu , and B. Han , “Unraveling the Mechanisms of Intervertebral Disc Degeneration: An Exploration of the p38 MAPK Signaling Pathway,” Frontiers in Cell and Developmental Biology 11 (2024): 1324561. 38313000 10.3389/fcell.2023.1324561 PMC10834758

[8] G. Bjørkøy , T. Lamark , A. Brech , et al., “p62/SQSTM1 Forms Protein Aggregates Degraded by Autophagy and Has a Protective Effect on Huntingtin‐Induced Cell Death,” Journal of Cell Biology 171 (2005): 603–614. 16286508 10.1083/jcb.200507002 PMC2171557

[9] J. Moscat and M. T. Diaz‐Meco , “p62 at the Crossroads of Autophagy, Apoptosis, and Cancer,” Cell 137 (2009): 1001–1004. 19524504 10.1016/j.cell.2009.05.023 PMC3971861

[10] T. Wang , Q. Jia , T. Chen , et al., “Alleviation of Synovial Inflammation of Juanbi‐Tang on Collagen‐Induced Arthritis and TNF‐Tg Mice Model,” Frontiers in Pharmacology 11 (2020): 45. 32116720 10.3389/fphar.2020.00045 PMC7033619

[11] Y. Takada , H. Ichikawa , V. Badmaev , and B. B. Aggarwal , “Acetyl‐11‐keto‐beta‐boswellic Acid Potentiates Apoptosis, Inhibits Invasion, and Abolishes Osteoclastogenesis by Suppressing NF‐Kappa B and NF‐Kappa B‐Regulated Gene Expression,” Journal of Immunology 176 (2006): 3127–3140. 10.4049/jimmunol.176.5.3127 16493072

[12] M. H. Pan , C. S. Lai , Y. J. Wang , and C. T. Ho , “Osthole Regulates Inflammatory Mediator Expression Through Modulating NF‐κB, Mitogen‐Activated Protein Kinases, Protein Kinase C, and Reactive Oxygen Species,” Journal of Agricultural and Food Chemistry 58 (2010): 10445–10451. 20839800 10.1021/jf102812t

[13] P. F. Hu , J. L. Tang , and W. E. Chen , “Osthole Improves Collagen‐Induced Arthritis in a Rat Model Through Inhibiting Inflammation and Cellular Stress,” Cellular and Molecular Biology Letters 23 (2018): 23. 29743895 10.1186/s11658-018-0086-0 PMC5932757

[14] W. Li , Y. Tang , Y. Chen , and J. A. Duan , “Advances in the Chemical Analysis and Biological Activities of Chuanxiong,” Molecules 17 (2012): 10614–10651. 22955453 10.3390/molecules170910614 PMC6268834

[15] J. Gao , X. Yin , N. Xu , T. Xue , and Z. Yang , “Mechanism of Ligusticum Cycloprolactam Against Neuroinflammation Based on Network Pharmacology and Experimental Verification,” Clinical and Experimental Pharmacology and Physiology 50 (2023): 729–742. 10.1111/1440-1681.13784 37308175

[16] K. Masuda , Y. Aota , C. Muehleman , et al., “A Novel Rabbit Model of Mild, Reproducible Disc Degeneration by an Anulus Needle Puncture,” Spine (Phila Pa 1976) 30 (2005): 5–14. 15626974 10.1097/01.brs.0000148152.04401.20

[17] C. W. A. Pfirrmann , A. Metzdorf , M. Zanetti , J. Hodler , and N. Boos , “Magnetic Resonance Classification of Lumbar Intervertebral Disc Degeneration,” Spine (Phila Pa 1976) 26 (2001): 1873–1878. 11568697 10.1097/00007632-200109010-00011

[18] J. P. Thompson , R. H. Pearce , M. T. Schechter , M. E. Adams , I. K. Tsang , and P. B. Bishop , “Preliminary Evaluation of a Scheme for Grading the Gross Morphology of the Human Intervertebral Disc,” Spine (Phila Pa 1976) 15 (1990): 411–415. 2363069 10.1097/00007632-199005000-00012

[19] M. V. Risbud and I. M. Shapiro , “Role of Cytokines in Intervertebral Disc Degeneration: Pain and Disc Content,” Nature Reviews Rheumatology 10 (2014): 44–56. 24166242 10.1038/nrrheum.2013.160 PMC4151534

[20] A. Jain , T. Lamark , E. Sjøttem , et al., “p62/SQSTM1 is a Target Gene for Transcription Factor NF‐κB and Promotes Neurite Outgrowth,” Journal of Cell Science 123 (2010): 2588–2597.

[21] L. Sanz , M. T. Diaz‐Meco , H. Nakano , and J. Moscat , “The Atypical Pkc‐Interacting Protein p62 Channels NF‐κB Activation by the IL‐1‐TRAF6 Pathway,” EMBO Journal 19 (2000): 1576–1586. 10747026 10.1093/emboj/19.7.1576 PMC310227

[22] E. Keil , R. Höcker , M. Schuster , et al., “Phosphorylation of Atg5 by the Gadd45β‐MEKK4‐p38 Pathway Inhibits Autophagy,” Cell Death and Differentiation 20 (2013): 321–332. 23059785 10.1038/cdd.2012.129 PMC3554344

[23] J. L. Webber and S. A. Tooze , “Coordinated Regulation of Autophagy by p38α MAPK Through mAtg9 and P38IP,” EMBO Journal 29 (2010): 27–40. 19893488 10.1038/emboj.2009.321 PMC2808369

[24] C. Zheng , X. Huang , Y. Li , M. Gong , and Y. Wang , “Osthole Ameliorates Cartilage Degradation by Downregulation of NF‐κB and HIF‐2α Pathways in an Osteoarthritis Murine Model,” International Immunopharmacology 79 (2020): 106145. 10.1016/j.ejphar.2019.172799 31765607

[25] H. Dong , L. He , M. Huang , and Y. Dong , “Osthole Inhibits M1 Macrophage Polarization and Attenuates Osteolysis in a Mouse Skull Model,” Oxidative Medicine and Cellular Longevity 2023 (2023): 4254972. 10.1155/2023/2975193 PMC9851800 36686380

[26] M. Abdel‐Tawab , “An Update on Pharmacological Potential of Boswellic Acids Against Chronic Diseases,” International Journal of Molecular Sciences 20 (2019): 4101. 31443458 10.3390/ijms20174101 PMC6747466

[27] M. L. Tiku , R. Shah , and G. T. Allison , “The Chondroprotective Effects of Ferulic Acid on Hydrogen Peroxide‐Stimulated Chondrocytes: Inhibition of Pro‐Inflammatory Cytokines and Metalloproteinase Gene Expression at the mRNA Level,” Inflammation Research 59 (2010): 561–567. 20349328 10.1007/s00011-010-0165-9

[28] G. Cai and H. Jiang , “Ferulic Acid Suppresses interleukin‐1β‐induced Degeneration of Chondrocytes Isolated From Patients With Osteoarthritis Through the SIRT1/AMPK/PGC‐1α Signaling Pathway,” Journal of Inflammation Research 14 (2021): 2455–2466. 10.1002/iid3.424 PMC8342228 34078001

[29] L. Zhao , J. Ye , G. T. Wu , X. J. Peng , P. F. Xia , and Y. Ren , “Gentiopicroside Prevents interleukin‐1 Beta Induced Inflammation Response in Rat Articular Chondrocyte,” Journal of Ethnopharmacology 172 (2015): 100–107. 26116164 10.1016/j.jep.2015.06.031

[30] Z. Xie , S. Zhang , Y. Zhou , et al., “Gentiopicroside Attenuates Collagen‐Induced Arthritis in Mice via Modulating the CD147/p38/NF‐κB Pathway,” Phytomedicine 102 (2022): 154141. 35598398 10.1016/j.intimp.2022.108854

[31] R. Tsujimoto , T. Yurube , Y. Takeoka , et al., “Involvement of Autophagy in the Maintenance of Rat Intervertebral Disc Homeostasis: An In‐Vitro and In‐vivo RNA Interference Study of Atg5,” Osteoarthritis and Cartilage 30 (2022): 481–493. 34958937 10.1016/j.joca.2021.12.004

[32] R. Kritschil , M. Scott , G. Sowa , and N. Vo , “Role of Autophagy in Intervertebral Disc Degeneration,” Journal of Cellular Physiology 237 (2022): 1266–1284. 34787318 10.1002/jcp.30631 PMC8866220

[33] H. Zhong , C. Yang , Y. Gao , et al., “PERK Signaling Activation Restores Nucleus Pulposus Degeneration by Activating Autophagy Under Hypoxia Environment,” Osteoarthritis and Cartilage 30 (2022): 341–353. 34767959 10.1016/j.joca.2021.11.005

