# Supplementary material for: Juanbi Tang Attenuates Intervertebral Disc Degeneration by Suppressing p38MAPK/NF‐κB Signaling and Activating Atg5‐Dependent Autophagy to Promote Macrophage M2 Polarization: In Vivo and In Vitro Evidence
Source: Immun Inflamm Dis. 2026 Sep 10;14(9):e70498. doi: 10.1002/iid3.70498 (PMC13560788; doi:10.1002/iid3.70498)
Supplement: Supplementary file 1 [file IID3-14-e70498-s001.docx]

# Supplementary Information

**Supplementary Fig. S1.** RT-qPCR mRNA quantification in primary rat NP cells across three groups: Control, LPS (1 μg/mL, 24 h), and LPS + JBT (50 μg/mL, 24 h). Targets: Bax, Bcl-2, LC3B, p62/SQSTM1, p38MAPK, NF-κB p65. Normalized to GAPDH. Mean ± SD, n = 3. *p < 0.05, **p < 0.01.

**Supplementary Fig. S2.** RT-qPCR mRNA quantification in RAW264.7 macrophages (nine experimental groups: normal, Atg5-OE and Atg5-KD × Control, LPS and LPS + JBT). Targets: Atg5, LC3B, p62/SQSTM1. Normalized to GAPDH. Mean ± SD, n = 3. *p < 0.05, **p < 0.01, ***p < 0.001.

| **Gene** | **Gene ID** | **Primer** | **Sequence (5'→3')** |
| --- | --- | --- | --- |
| Bax | 24887 | Forward | TGG CTT CAG GGT TTC ATC CAG |
|  |  | Reverse | GTC CCG AAG TAG GAG AGG CA |
| Bcl-2 | 24224 | Forward | ATG CGT GAC GAC TTC TCC AC |
|  |  | Reverse | CAG ACA GCC AGG AGA AAT CAA |
| LC3B | 64862 | Forward | CAC TGC TCT GTC TTG TGT AGG TTG |
|  |  | Reverse | TCG TTG TGC CTT TAT TAG TGC ATC |
| p62/SQSTM1 | 113894 | Forward | AGA TGC CAG AAT CGG AAG GGA |
|  |  | Reverse | CTG AAA CTC CAG TTG TCC CAG |
| p38 MAPK | 81649 | Forward | ATG GAG CAG TTC AGC AAG CAC |
|  |  | Reverse | CTT GGT GAA CTC GTT GAT GGC |
| NF-κB p65 | 309165 | Forward | ATG TGG AGA TCA TTG AGC AGC |
|  |  | Reverse | CTT GTC TGT GGA GCA AAG GCT |
| Atg5 | 365601 | Forward | AAA GAT GTG CTT CGA GAT GTG TC |
|  |  | Reverse | CAC TTT GTC AGT TAC CAA CGT CA |
| GAPDH | 24383 | Forward | GGC AAG TTC AAC GGC ACA GT |
|  |  | Reverse | CGC CAG TAG ACT CCA CGA CAT |

**Supplementary Table S1. Real-time PCR primer sequences used in this study.**

All primers are designed for rat genes and validated for specificity by NCBI Primer-BLAST. GAPDH served as the internal reference gene for the RT-qPCR experiments reported in Supplementary Figs. S1 and S2.

| **Latin name** | **Pinyin name** | **Depository** |
| --- | --- | --- |
| *Notopterygium incisum Ting ex H.T. Chang* | Qianghuo | Herbarium, Chengdu University of Traditional Chinese Medicine |
| *Angelica pubescens Maxim. f. biserrata Shan et Yuan* | Duhuo | Herbarium, Chengdu University of Traditional Chinese Medicine |
| *Cinnamomum cassia Presl* | Rougui | Herbarium, Chengdu University of Traditional Chinese Medicine |
| *Gentiana macrophylla Pall.* | Qinjiao | Herbarium, Chengdu University of Traditional Chinese Medicine |
| *Ligusticum chuanxiong Hort.* | Chuanxiong | Herbarium, Chengdu University of Traditional Chinese Medicine |
| *Angelica sinensis (Oliv.) Diels* | Danggui | Herbarium, Chengdu University of Traditional Chinese Medicine |
| *Piper kadsura (Choisy) Ohwi* | Haifengteng | Herbarium, Chengdu University of Traditional Chinese Medicine |
| *Morus alba L.* | Sangzhi | Herbarium, Chengdu University of Traditional Chinese Medicine |
| *Aucklandia lappa Decne.* | Muxiang | Herbarium, Chengdu University of Traditional Chinese Medicine |
| *Boswellia sacra Flueck.* | Ruxiang | Herbarium, Chengdu University of Traditional Chinese Medicine |
| *Glycyrrhiza uralensis Fisch. ex DC.* | Gancao | Herbarium, Chengdu University of Traditional Chinese Medicine |

**Supplementary Table S2. Voucher specimen information for Juanbi Tang herbal components.**

All medicinal herbs were authenticated against reference specimens in accordance with the 2020 Chinese Pharmacopoeia. Voucher specimens are deposited at the Herbarium of Chengdu University of Traditional Chinese Medicine, Chengdu, China.
